# Supplementary material for: Greater Adherence to Dietary Guidelines Associated with Reduced Risk of Cardiovascular Diseases in Chinese Patients with Type 2 Diabetes
Source: Nutrients. 2022 Apr 20;14(9):1713. doi: 10.3390/nu14091713 (PMC9103846; doi:10.3390/nu14091713)
Supplement: Supplementary file 1 [file nutrients-14-01713-s001.zip › nutrients-1634189-supplementary.pdf]

Online Supplemental Table S1. Components and scoring standards of HEI-2015 and CHEI scores with the standard cup/ounce equivalents and standard portion from MPED and DGC-2016 [a]

	HEI-2015 [b]		CHEI	
	**Maximum points and standard for maximum score**	**Minimum points and standard for minimum score of zero**	**Maximum points and standard for maximum score**	**Minimum points and standard for minimum score of zero**
Component	100 points total (13 components: 5-10 points each)		100 points total (17 components: 5-10 points each)	
**Adequacy**				
Fruits	Total fruits 5 points: ≥0.8 cup equiv.	0 point: no fruit	Total fruits 10 points: ≥1.1 SP	0 point: no fruit
	Whole fruits [c] 5 points: ≥0.4 cup equiv.	0 point: no whole fruit		
Vegetables	Total vegetables 5 points: ≥1.1 cup equiv.	0 point: no vegetables	Total vegetables 5 points: ≥1.9 SP	0 point: no vegetables
	Greens and beans 5 points: ≥0.2 cup equiv.	0 point: no greens or beans	Dark vegetables 5 points: ≥0.9 SP	0 point: no dark vegetables
Grains	Whole grains 10 points: ≥1.5 oz equiv.	0 point: no whole grains	Total grains 5 points: ≥2.5 SP	0 point: no grains
			Whole grains and mixed beans [d] 5 points: ≥0.6 SP	0 point: no whole grains or mixed beans
			Tubers 5 points: ≥0.3 SP	0 point: no tubers
Dairy	10 points: ≥1.3 cup equiv.	0 point: no diary	5 points: ≥0.5 SP	0 point: no diary
Protein foods	Total protein foods 5 points: ≥2.5 oz equiv.	0 point: no protein foods	Soybeans 5 points: ≥0.4 SP	0 point: no soybeans
	Seafood and plant proteins 5 points: ≥0.8 oz equiv.	0 point: no seafood or plant proteins	Fish and seafood 5 points: ≥0.6 SP	0 point: no fish or seafood
			Poultry 5 points: ≥0.3 SP	0 point: no poultry

			Eggs 5 points: ≥0.5 SP	0 point: no eggs
Fats	(PUFAs + MUFAs)/SFAs ratio 10 points: ≥2.5	0 point: ≤1.2	Seeds and nuts 5 points: ≥0.4 SP	0 point: no seeds and nuts
**Moderation**				
Refined grains	10 points: ≤1.8 oz equiv.	0 point: ≥4.3 oz equiv.		
Red meats			5 points: ≤0.4 SP	0 point: ≥3.5 SP
Sodium	10 points: ≤1.1 grams	0 point: ≥2.0 grams	10 points: ≤1000 mg	0 point: ≥3608 mg
Added sugar	10 points: ≤6.5% of energy	0 point: ≥26% of energy	5 points; ≤10% of energy	0 point: ≥20% of energy
Oils/fats	Saturated fats 10 points: ≤8% of energy	0 point: ≥16% of energy	Cooking oils 10 points: ≤15.6 g	0 point: ≥32.6 g
Alcohol			5 points: ≤60 g (men)/40 g (women)	0 point: ≥25 g (men)/15 g (women)

Abbreviations: HEI-2015, Healthy Eating Index-2015; CHEI, Chinese Healthy Eating Index; MPED, MyPyramid Equivalents Database; oz, ounce; equiv., equivalent; DCG-2016, Dietary Guidelines for Chinese Residents; SP, standard portion; PUFA, polyunsaturated fatty acids; MUFA, monounsaturated fatty acids; SFA, saturated fatty acids.

[a] Food consumption and scores are expressed as cup/ounce equivalents from MPED: 1 cup equivalent=225ml, 1 oz equivalent=28.3g; foods consumption and scores are expressed as standard portion from DGC-2016, SP in one food group shares similar energy and contents of carbohydrates, fats, and protein;

[b] Using energy density approach: all components consumption except (PUFAs + MUFAs)/SFAs ratio and alcohol are per 1000kcal.

[c] Including all forms fruit except fruit juice.

[d] Mixed beans include mung bean, red bean, broad bean, pea and kidney bean, etc.
